# The benefit and risk of nivolumab in non‐small‐cell lung cancer: a single‐arm meta‐analysis of noncomparative clinical studies and randomized controlled trials

**DOI:** 10.1002/cam4.1387

**Published:** 2018-03-23

**Authors:** Binghao Zhao, Wenxiong Zhang, Dongliang Yu, Jianjun Xu, Yiping Wei

**Affiliations:** ^1^ Department of thoracic surgery The second affiliated hospital of Nanchang University Nanchang China

**Keywords:** Nivolumab, non‐small‐cell lung cancer, single‐arm meta‐analysis

## Abstract

Nivolumab is a programmed cell death 1 (PD‐1) receptor inhibitor antibody that enhances immune system antitumor activity. Although it is used for treating advanced non‐small‐cell lung cancer (NSCLC), its actual efficacy has not been determined. We searched PubMed, the Cochrane Library, Embase, MEDLINE, and Web of Science for related noncomparative clinical studies and randomized controlled trials (RCTs) to assess nivolumab benefit and risk in NSCLC. The main outcomes were objective response rate (ORR), 1‐year overall survival rate (1‐yOS rate), and progression‐free survival rate at 24 weeks (PFS at 24 weeks rate), any‐grade adverse effects rate (any‐grade AEs%), and grade 3–4 AE rate (grade 3–4 AEs%). Relative risk (RR) was used to compare ORR in patients with positive and negative programmed cell death ligand 1 (PD‐L1) expression. Random‐effects models were used to determine pooled effect size and two‐sided 95% confidence intervals (95% CI). We included 20 studies (17 noncomparative open‐label cohort studies, three RCTs) involving 3404 patients in our meta‐analysis. The modified nivolumab ORR was 18% (95% CI: 15–20%), the 1‐yOS rate was 45% (95% CI: 40–50%), PFS at 24 weeks rate was 42% (95% CI: 37–48%), any‐grade AEs% was 61% (95% CI: 50–73%), and grade 3–4 AEs% was 12% (95% CI: 9–16%). PD‐L1 expression was related with the nivolumab ORR. Nivolumab potentially causes ongoing response, long‐term PFS, and reduced treatment‐related AEs. PD‐L1 expression predicts the outcome of nivolumab immunotherapy. More high‐quality and well‐designed RCTs with large sample sizes are warranted to prove our findings.

## Introduction

Lung cancer is the leading cause of cancer‐related mortality worldwide, with non‐small‐cell lung cancer (NSCLC) accounting for approximately 80–85% of all cases. Most patients with advanced NSCLC have poor prognosis, where the 5‐year survival rate is <5% 1. Checkpoints are specific molecules on T cells and antigen‐presenting cells 2, when tumor cell activation of the signaling pathways downregulates the T‐cell immune response. In this context, checkpoint inhibitors are new, emerging, potential strategies in oncology, especially in advanced NSCLC. Ipilimumab is a monoclonal inhibitor antibody directed against cytotoxic T‐lymphocyte‐associated antigen 4 (CTLA‐4), a known checkpoint, that has shown exciting effects on melanoma and lung cancer 3. Programmed cell death 1 (PD‐1) and programmed cell death ligand 1 (PD‐L1) may also demonstrate extensive antitumor activity 4, 5. Nivolumab is a fully human immunoglobulin 4 (IgG4) PD‐1 receptor inhibitor antibody that was approved in 2015 by the US Food and Drug Administration for treating melanoma, renal cell carcinoma, and NSCLC with durable response and tolerable toxicity. Besides, nivolumab reserves preferable option for classical Hodgkin's lymphoma, head and neck squamous cell carcinoma (HNSCC), and urothelial cancer 6.

The pharmaceutical giant Bristol‐Myers Squibb (BMS) provided the main sponsorship for producing this novel monoclonal antibody; however, many single‐arm studies have reported the benefits and risks of nivolumab for treating NSCLC without control therapies, and the results reported were controversial. The small sample sizes were not adequately powered to detect the actual efficacy of nivolumab and might misestimate its performance in clinical practice. Hence, we systematically reviewed the current available literature to conduct the present single‐arm meta‐analysis with the aim of describing the general benefit and risk of nivolumab. The secondary objective was to evaluate whether the objective response rate (ORR) of nivolumab‐treated patients with NSCLC with positive and negative PD‐L1 expression is significantly different.

## Materials and Methods

Our single‐arm meta‐analysis is accordance with PRISMA (Preferred Reporting Items for Systematic Review and Meta‐Analysis) guidelines (Table S1) 7 and has been registered with PROSPERO (International Prospective Register of Systematic Reviews, CRD42017064411).

### Search strategy

We systematically searched PubMed, the Cochrane Library, Embase, MEDLINE, and Web of Science from 1 January 2012 to 31 December 2017 without any language restrictions for noncomparative clinical studies and randomized controlled trials (RCTs). The combined text and medical subject heading (MeSH) terms used were as follows: “Carcinoma, Non‐Small‐Cell Lung” and “nivolumab.” The complete search we used for PubMed was as follows: (Carcinoma, Non‐Small‐Cell Lung [MeSH terms] OR Carcinoma, Non Small Cell Lung [text] OR Carcinomas, Non‐Small‐Cell Lung [text] OR Lung Carcinoma, Non‐Small‐Cell [text] OR Lung Carcinomas, Non‐Small‐Cell [text] OR Non‐Small‐Cell Lung Carcinomas [text]) AND (nivolumab [MeSH term] OR MDX‐1106 [text] OR ONO‐4538 [text] OR BMS‐936558 [text] OR Opdivo [text]). We also manually searched the reference lists of the retrieved literature for further eligible articles.

### Selection criteria

We included studies that met the following criteria 8: (1) Adult patients with advanced NSCLC whose life expectancy was at least 3 months and without any autoimmune disease; Eastern Cooperative Oncology Group performance status was ≤2; there were no restrictions and no significant difference on sex, race, region, nationality, pretreatment; (2) Single‐agent nivolumab or in combination with other chemotherapy drugs; (3) Whether comparison had been performed; (4) The main study outcome directly or indirectly included ORR, 1‐year overall survival rate (1‐yOS rate), progression‐free survival rate at 24 weeks (PFS at 24 weeks rate), any‐grade adverse effects rate (any‐grade AEs%), and grade 3–4 adverse effects rate (grade 3–4 AEs%; treatment‐related AE status was assessed using the Lung Cancer Symptom Scale and the European Quality of Life–5 Dimensions questionnaire 9); and (5) Noncomparative clinical studies (noncomparative open‐label studies) and RCTs.

The most complete and novel reports were included for data extraction and assessments if the objects were duplicated. We excluded reviews without original data, meta‐analyses, and animal experiments.

### Data extraction and quality assessment

Two investigators (W.X. Zhang and D.L. Yu) extracted the following information from each included study independently: first author, publication year, region, number of participants enrolled, participant characteristics, phase of clinical study, cohort completeness, tumor histology, clinical setting, endpoint, corresponding provided outcome, and study design. For noncomparative studies, we extracted total summarized data and subgroup figures or information if available; from the RCTs, we extracted data on clinical setting and outcome of intervention and control groups. The main outcomes were ORR, 1‐yOS rate, PFS at 24 weeks rate, any‐grade AEs%, and grade 3–4 AEs%. The other outcomes included median OS (mOS, months), median PFS (mPFS, months), median duration of response (mDOR, weeks), complete response rate (CR), partial response rate (PR), stable disease rate (SD), disease control rate (DCR), and ongoing response rate. All extracted information and original data were entered in standardized tables. Although not treated as a main outcome, we report the detailed AE conditions and PD‐L1 biomarker status. Disagreements were resolved by a third investigator (Y.P. Wei).

We used the Cochrane risk of bias tool 10 to evaluate the quality of eligible RCTs. However, there are no textbook quality guidelines for noncomparative clinical studies, for which there is large heterogeneity, to date 11. Therefore, we only assessed study quality via powered data volume and integrity, distinguished journals, and influential writers or teams who represented the leading position of NSCLC course and research.

### Statistical analysis

For the included studies, we analyzed the main outcomes only concerning nivolumab specifically. We input the total clinical setting percentage for the main outcome and number of participants of each study, and then calculated the corresponding standard errors of these quasinormal distribution “rates” using Stata (StataCorp, USA). The 95% confidence lower interval (LI) and upper interval (UI) derived from the “rates” and standard errors could be justified. Lastly, the pooled effect sizes (ES), which denoted median “rates” and the 95% confidence intervals (95% CI), were output 12. We also modified the final ES by omitting studies with large variability. Pooled ES aided the general evaluation of nivolumab benefit and risk. Heterogeneity across studies was examined by the Cochran *Q* chi‐square test and the *I* ^2^ statistic. Studies with an *I* ^2^ statistic of 25–50%, 50–75%, and >75% were deemed to have low, moderate, and high heterogeneity, respectively 13. *P *<* *0.1 for the *Q* test was taken to indicate significant heterogeneity. We used random‐effects models for all pooled ES because there was great subjectivity given the lack of related control groups in the noncomparative studies, and a tendency toward high heterogeneity 14. Subgroup analyses were conducted according to study design, medication type, program subgroup, region, study phase, and histology. The subgroup analyses were performed only for modified ORR and grade 3–4 AEs% because of the abundant available data.

Relative risk (RR) and the random‐effects model were used to estimate whether there was a significant difference in ORR between patients with positive and negative PD‐L1 expression.

Potential publication bias among the main outcomes was assessed using the Egger linear regression test 15. All analyses were performed using Stata statistical software version 12.0 (StataCorp, USA, https://www.stata.com); two‐sided *P *<* *0.05 was considered statistically significant except where otherwise specified.

## Results

### Study identification

**Figure 1:**
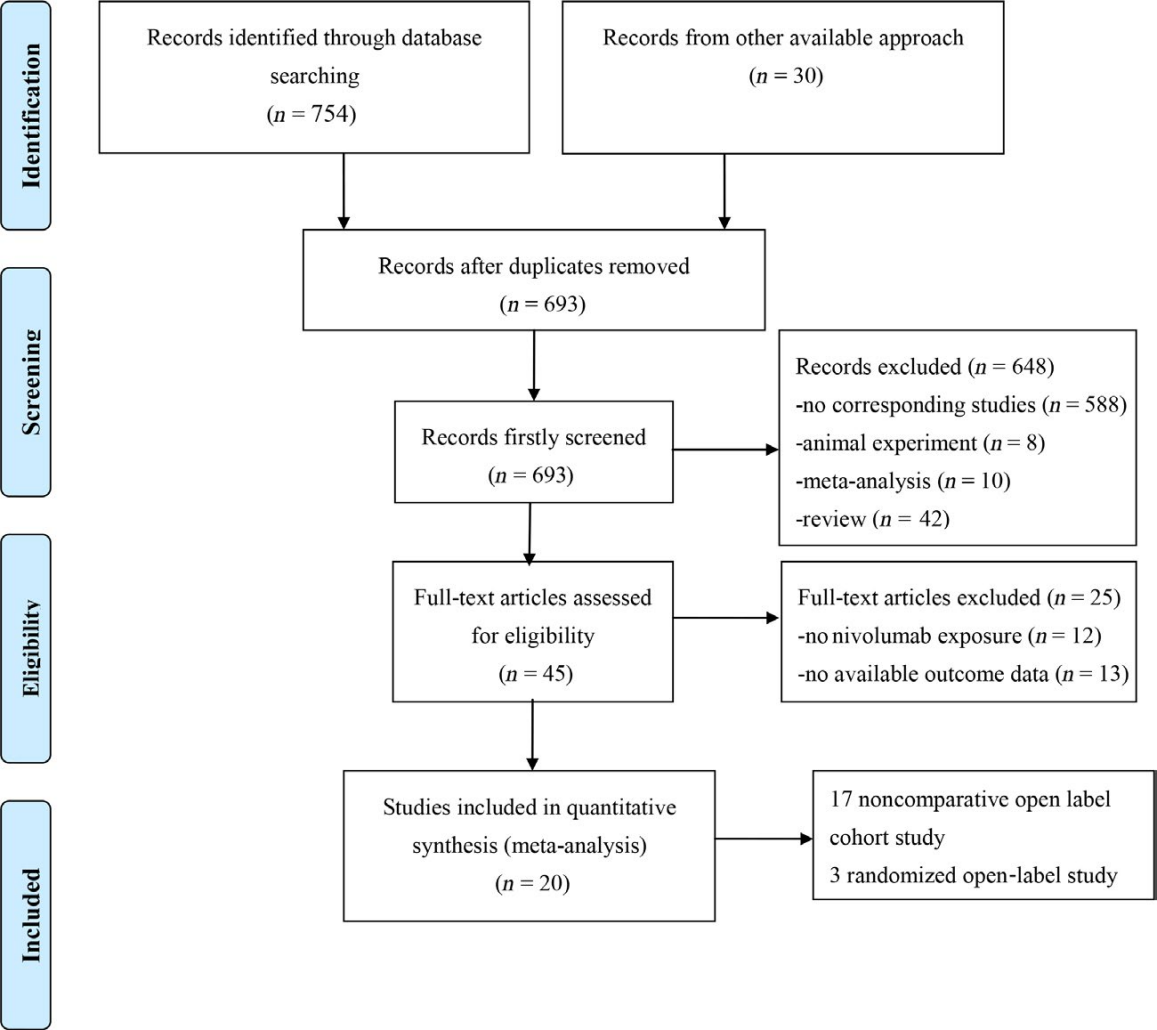
Flow chart of the single‐arm meta‐analysis.

Searching the above‐mentioned databases yielded a total 754 records, and we obtained another 30 records from the reference lists and Google Scholar. We excluded 61 articles for duplication, 648 studies were excluded for next exclusion. The sequence order step and quantity of next exclusion was (1) 588 studies were excluded for no corresponding studies (*n* = 588), (2) review (*n* = 42), (3) animal experiment (*n* = 8), and (4) meta‐analysis (*n* = 10). The remaining 45 studies were assessed through full‐text revision, and 25 of them were excluded because of two reasons: first no nivolumab exposure (*n* = 12) and second no available outcome data (*n* = 13). The remaining 20 articles were eligible for quantitative synthesis. Figure 1 shows the selection process.

### Study characteristics

The present single‐arm meta‐analysis included a total 20 studies 16, 17, 18, 19, 20, 21, 22, 23, 24, 25, 26, 27, 28, 29, 30, 31, 32, 33, 34, 35 involving 3404 participants; Table 1 describes the main study characteristics, and Table 2 presents the outcome results. The studies were all published between 2012 and 2017. The sample sizes of each study ranged from 33 to 582 (of a total 3404, there were 2754 patients in the nivolumab group for main outcome calculation). Three studies 23, 24, 35 were randomized, open‐label studies (CheckMate 017, CheckMate 057; CheckMate 026, unblinded), and 17 studies 16, 17, 18, 19, 20, 21, 22, 33, 34 were noncomparative, open‐label cohort studies. Nine studies 17, 18, 19, 20, 21, 22, 26, 28, 30 were published abstracts, and eleven studies 16, 23, 24, 25, 27, 29, 31, 32, 33, 34, 35 were original studies. Three studies 29, 30, 31 had been performed in Europe; the remaining 17 16, 17, 18, 19, 20, 21, 22, 23, 24, 25, 26, 27, 31, 32, 33, 34, 35 had been conducted in North America. Eight studies 16, 17, 18, 19, 21, 25, 32, 33 set nivolumab subgroups based on dosage or cycle; six studies 17, 18, 19, 21, 32, 33 used nivolumab concurrently with chemotherapy drugs (platinum‐based 17, 32, ipilimumab 18, 19, 33, bevacizumab 21). There were 10 phase I studies 16, 17, 18, 19, 21, 22, 25, 31, 32, 33, four phase II studies 20, 26, 27, 34, and six phase III studies 23, 24, 28, 29, 30, 35. Five studies 20, 24, 27, 28, 30 included patients with squamous NSCLC for clinical study; two studies 23, 34 involved patients with nonsquamous NSCLC; and 13 studies 16, 17, 18, 19, 21, 22, 25, 26, 29, 31, 32, 33, 35 involved mixed histological types. Eight studies reported the ORR in positive and negative PD‐L1 expression. All studies were published in English, and all enrolled patients had received prior relevant therapy.

**Table 1:** Characteristics of clinical studies included in the single‐arm meta‐analysis

Study		Region	Patients	Age (years)	Male (%)	Quit	Phase	Tumor histology	Drug	Clinical setting	Combined with	Study design
2012	Topalian et al. 16	North America	76	NR	NR	NR	I	Squamous (*n* = 18)	Nivolumab 1 mg/kg (*n* = 18)	Q2W, IV total 12 cycles	NR	Noncomparative open‐label cohort study
								Nonsquamous (*n* = 56)	Nivolumab 3 mg/kg (*n* = 19)	Q2W, IV total 12 cycles		
								Unknown (*n* = 2)	Nivolumab 10 mg/kg (*n* = 39)	Q2W, IV total 12 cycles		
2013	Rizvi et al. 17	North America	43	NR	NR	3	I	Squamous (*n* = 15)	Nivolumab 10 mg/kg (*n* = 12)^a^	Q3W, IV	Gemcitabine, cisplatin, pemetrexed, carboplatin, paclitaxel^c^	Noncomparative open‐label cohort study
								Nonsquamous (*n* = 28)	Nivolumab 10 mg/kg (*n* = 15)	Q3W, IV^b^		
									Nivolumab 10 mg/kg (*n* = 16)	Q3W, IV		
2014	Antonia et al. 18	North America	49	NR	NR	18	I	Squamous (*n* = 18)	Nivolumab 1 mg/kg (*n* = 24)	Q3W, IV for 4 cycles followed by nivolumab 3 mg/kg, Q2W, IV	Ipilimumab^d^	Noncomparative open‐label cohort study
								Nonsquamous (*n* = 31)	Nivolumab 3 mg/kg (*n* = 25)	Q3W, IV for 4 cycles followed by nivolumab 3 mg/kg, Q2W, IV		
2014	Antonia et al. 19	North America	46	NR	NR	16	I	Squamous (*n* = 15)	Nivolumab 1 mg/kg (*n* = 22)	Q3W, IV for 4 cycles followed by nivolumab 3 mg/kg, Q2W, IV	Ipilimumab	Noncomparative open‐label cohort study
								Nonsquamous (*n* = 31)	Nivolumab 3 mg/kg (*n* = 24)	Q3W, IV for 4 cycles followed by nivolumab 3 mg/kg, Q2W, IV		
2014	Ramalingam et al. 20	North America	117	65 (37–87)^e^	73	11	II	Squamous (*n* = 117)	Nivolumab 3 mg/kg (*n* = 117)	Q2W, IV	NR	Noncomparative open‐label cohort study
2014	Rizvi et al. 21	North America	33	NR	NR	NR	I	Squamous (*n* = 13)	Nivolumab 5 mg/kg (*n* = 12)	Q3W, IV	Bevacizumab^a^ (BEV)	Noncomparative open‐label cohort study
								Nonsquamous (*n* = 20)	Nivolumab 3 mg/kg (*n* = 21)	Q3W, IV		
2015	Bauer et al. 22	North America	226	67 (33–91)	55	47	I	Squamous (*n* = 59)	Nivolumab 3 mg/kg (*n* = 226)	Q2W, IV	NR	Noncomparative open‐label cohort study
								Nonsquamous (*n* = 167)				
2015	Borghaei et al. 23	North America	292	61 (37–84)	52	15	III	Nonsquamous (*n* = 582)	Nivolumab 3 mg/kg (*n* = 292)	Q2W, IV	NR	Randomised open‐label study
			290	64 (21–85)	58	44			Docetaxel 75 mg/m^2^ (*n* = 290)	Q3W, IV		
2015	Brahmer et al. 24	North America	135	62 (39–85)	82	4	III	Squamous (*n* = 272)	Nivolumab 3 mg/kg (*n* = 135)	Q2W, IV	NR	Randomised open‐label study
			137	64 (42–84)	71	14			Docetaxel 75 mg/m^2^ (*n* = 137)	Q3W, IV		
2015	Gettinger et al. 25	North America	129	65 (38–85)	79	18	I	Squamous (*n* = 54)	Nivolumab 1 mg/kg (*n* = 33)	Q2W, IV for 12 cycles	NR	Noncomparative open‐label cohort study
								Nonsquamous (*n* = 74)	Nivolumab 3 mg/kg (*n* = 37)	Q2W, IV for 12 cycles		
								Unknown (*n* = 1)	Nivolumab 10 mg/kg (*n* = 59)	Q2W, IV for 12 cycles		
2015	Nishio et al. 26	North America	111	NR	NR	NR	II	Squamous (*n* = 35)	Nivolumab 3 mg/kg (*n* = 111)	Q2W, IV	NR	Noncomparative open‐label cohort study
								Nonsquamous (*n* = 76)				
2015	Rizvi et al. 27	North America	117	65 (57–61)	85	14	II	Squamous (*n* = 117)	Nivolumab 3 mg/kg (*n* = 117)	Q2W, IV	NR	Noncomparative open‐label cohort study
2016	Bidoli et al. 28	Europe	372	NR	NR	NR	III	Squamous (*n* = 372)	Nivolumab 3 mg/kg (*n* = 372)	Q2W, IV for 12 cycles	NR	Noncomparative open‐label cohort study
2016	Brustugun et al. 29	Europe	58	64.6 (32–88)	48	4	III	Squamous (*n* = 24)	Nivolumab 3 mg/kg (*n* = 58)	IV	NR	Noncomparative open‐label cohort study
								Nonsquamous (*n* = 34)				
2016	Crino et al. 30	Europe	371	NR	22	NR	III	Squamous (*n* = 371)	Nivolumab 3 mg/kg (*n* = 371)	Q2W, IV for 12 cycles	NR	Noncomparative open‐label cohort study
2016	Gettinger et al. 31	North America	52	67 (43–85)	50	6	I	Squamous (*n* = 13))	Nivolumab 3 mg/kg (*n* = 52)	Q2W, IV	NR	Noncomparative open‐label cohort study
								Nonsquamous (*n* = 39)				
2016	Rizvi et al. 32	North America	56	64 (34–83)	46	12	I	Squamous (*n* = 16)	Nivolumab 10 mg/kg^a^ (*n* = 12)	Q3W, IV for 4 cycles	Gemcitabine, cisplatin, pemetrexed, paclitaxel, carboplatin	Noncomparative open‐label cohort study
								Nonsquamous (*n* = 37)	Nivolumab 10 mg/kg (*n* = 15)	Q3W, IV for 4 cycles		
								Unknown (*n* = 3)	Nivolumab 10 mg/kg (*n* = 15)	Q3W, IV for 4 cycles		
									Nivolumab 10 mg/kg (*n* = 14)	Q3W, IV for 4 cycles		
2017	Hellmann et al. 33	North America	77	NR	10	10	I	Squamous (*n* = 13)	Nivolumab 3 mg/kg^f^ (*n* = 38)	Q2W, IV	Ipilimumab	Noncomparative open‐label cohort study
								Nonsquamous (*n* = 64)	Nivolumab 3 mg/kg (*n* = 39)	Q2W, IV		
2016	Nisho et al. 34	North America	76	64 (39–78)	65	12	II	Nonsquamous (*n* = 76)	Nivolumab 3 mg/kg (*n* = 76)	Q2W, IV	NR	Noncomparative open‐label cohort study
2017	Carbone et al. 35	North America	270	63 (32–89)	68	27	III	Squamous (*n* = 129)	Nivolumab 3 mg/kg (*n* = 270)	Q2W, IV for 6 cycles	NR	Randomised open‐label study
			271	65 (29–87)	55	35		Nonsquamous (*n* = 412)	Platinum‐based (*n* = 271)^c^	Q3W, IV for 6 cycles		

Q2W, every 2 weeks; Q3W, every 3 weeks; IV, intravenous.

a The same dosage and duration of nivolumab with different types of platinum‐based doublet chemotherapy.

b Complete response or progressive disease or unacceptable toxicity or achieved total medication cycles are all the endpoints.

c Platinum‐based doublet chemotherapy (gemcitabine, cisplatin, pemetrexed, carboplatin, and paclitaxel).

d Ipilimumab is a recombinant, fully human, monoclonal antibody targeted at cytotoxic T‐lymphocyte‐associated antigen 4 (CTLA‐4) that is available for the treatment of advanced melanoma.

e Median age (range).

f The same dosage and duration of nivolumab with different types of ipilimumab.

**Table 2:** Original data extracted from included studies

Study		Patients	Main outcome					Other outcome							
			ORR (%)	1‐yOS rate %	PFS at 24‐week rate %	Any‐grade AEs %	Grade 3–4 AEs %	mOS months (Range)	mPFS months (Range)	mDOR weeks (Range)	CR %	PR %			
2012	Topalian et al. 16	Total (*n* = 76)	18.4	NR	26.3	NR	NR	NR	NR	(3.7–30.8^+^)^a^	NR	NR	6.6^b^	NR	NR
		Arm A (*n* = 18)	5.6	NR	16.7	NR	NR	NR	NR	NR	NR	NR	5.6	NR	NR
		Arm B (*n* = 19)	31.6	NR	42.1	NR	NR	NR	NR	NR	NR	NR	10.5	NR	NR
		Arm C (*n* = 39)	17.9	NR	23.1	NR	NR	NR	NR	NR	NR	NR	5.1	NR	NR
2013	Rizvi et al. 17	Total (*n* = 43)	32.3	NR	NR	NR	48.8	NR	NR	NR	NR	NR	32.7	NR	NR
		Arm A (*n* = 12)	33.0	NR	NR	NR	25.0	NR	NR	NR	NR	NR	NR	NR	NR
		Arm B (*n* = 15)	33.0	NR	NR	NR	46.7	NR	NR	NR	NR	NR	NR	NR	NR
		Arm C (*n* = 16)	31.0	NR	NR	NR	68.8	NR	NR	NR	NR	NR	NR	NR	NR
2014	Antonia et al. 18	Total (*n* = 49)	16.0	NR	41.3	NR	49.0	NR	NR	(6.1^+^–49.1^+^)	NR	NR	NR	NR	NR
		Arm A (*n* = 24)	12.5	NR	28.6	NR	NR	NR	NR	NR	NR	NR	NR	NR	NR
		Arm B (*n* = 25)	20.0	NR	NR	NR	NR	NR	NR	NR	NR	NR	NR	NR	NR
2014	Antonia et al. 19	Total (*n* = 46)	22.0	NR	NR	84.8	44.9	NR	NR	NR	NR	NR	32.6	NR	8.7
		Arm A (*n* = 22)	13.6	NR	NR	NR	NR	NR	NR	NR	NR	NR	36.4	NR	9.1
		Arm B (*n* = 24)	29.2	NR	NR	NR	NR	NR	NR	NR	NR	NR	29.2	NR	8.3
2014	Ramalingam et al. 20	Total (*n* = 117)	12.0	NR	27.0	71.0	15.0	NR	1.9	NR	NR	NR	29.1	NR	NR
2014	Rizvi et al. 21	Total (*n* = 33)	9.1	NR	NR	72.7	25.0	NR	NR	NR	NR	NR	48.5	NR	18.2
		Arm A (*n* = 12)	8.3	75.0	NR	91.7	25.0	(33.3–86.7^+^)	NR	NR	NR	NR	50.0	NR	15.7
		Arm B (*n* = 21)	9.5	NR	NR	61.9	25.0	(2.1–56.3)	NR	NR	NR	NR	47.6	NR	18.3
2015	Bauer et al. 22	Total (*n* = 226)	13.7	NR	NR	13.3	1.8	NR	NR	NR	0	13.7	43.1	NR	NR
2015	Borghaei et al. 23	Total (*n* = 582)	15.8	45.0	NR	78.1	31.9	NR	NR	NR	NR	NR	NR	NR	NR
		Nivolumab (*n* = 292)	19.2	51.0	NR	68.2	10.3	12.2 (9.7–15.0)^c^	2.3 (2.2–3.3)	17.2 (1.8–22.6^+^)	1.4	17.8	25.3	NR	51.8
		Docetaxel (*n* = 290)	12.4	39.0	NR	88.1	53.7	9.4 (8.1–10.7)	4.2 (3.5–3.9)	5.6 (1.2^+^–15.2^+^)	0.3	12.1	42.1	NR	13.9
2015	Brahmer et al. 24	Total (*n* = 272)	14.3	33.1	NR	71.9	30.8	NR	NR	NR	NR	NR	NR	NR	NR
		Nivolumab (*n* = 135)	20.0	42.2	NR	58.0	6.9	9.2 (7.3–13.3)	3.5 (2.1–4.9)	(2.9–20.5^+^)	0.7	19.3	28.9	NR	63.0
		Docetaxel (*n* = 137)	8.8	24.1	NR	86.0	55.0	6.0 (5.1–7.3)	2.8 (2.1–3.5)	8.4 (1.4^+^–15.2^+^)	0	8.8	34.3	NR	33.3
2015	Gettinger et al. 25	Total (*n* = 129)	17.1	41.9	NR	41.1	14.0	9.9 (7.8–12.4)	NR	17.0 (1.4^+^–36.8^+^)	NR	NR	NR	NR	NR
		Arm A (*n* = 33)	3.0	33.3	NR	NR	NR	9.2 (5.3–11.1)	NR	14.7 (14.7–14.7)	NR	NR	NR	NR	NR
		Arm B (*n* = 37)	24.3	56.8	NR	NR	NR	14.9 (7.3–30.3)	NR	17.0 (3.7^+^–32.6^+^)	NR	NR	NR	NR	NR
		Arm C (*n* = 59)	20.3	37.3	NR	NR	NR	9.2 (5.2–12.4)	NR	19.1 (1.4^+^–36.8^+^)	NR	NR	NR	NR	NR
2015	Nishio et al. 26	Total (*n* = 111)	21.6	NR	NR	NR	16.2	NR	NR	4.3^b^	NR	NR	NR	NR	NR
2015	Rizvi et al. 27	Total (*n* = 117)	14.5	39.3	NR	74.4	17.1	NR	1.9 (1.8–3.2)	(8.31‐)	0.9	12.0	25.6	76.5	NR
2016	Bidoli et al. 28	Total (*n* = 372)	4.0	NR	NR	NR	NR	NR	NR	NR	0.6	3.4	6.3	47.3	NR
2016	Brustugun et al. 29	Total (*n* = 58)	NR	50.0	NR	31.0	5.2	11.7	NR	NR	NR	NR	NR	NR	NR
2016	Crino et al. 30	Total (*n* = 371)	18.1	NR	NR	28.6	5.4	9.1 (6.7–11.5)	3.9 (3.2–4.6)	NR	0.8	16.7	29.4	47.0	NR
2016	Gettinger et al. 31	Total (*n* = 52)	23.1	73.1	40.4	71.2	19.2	19.4 (0.2–35.8^+^)	3.6 (<0.1^+^–28^+^)	(4.2–25.8^+^)	NR	NR	26.9	50.0	66.7
2016	Rizvi et al. 32	Total (*n* = 56)	42.9	71.4	53.4	94.6	44.6	NR	NR	NR	1.8	41.1	42.9	85.7	41.7
		Arm A (*n* = 12)	33.3	50.0	50.0	NR	NR	11.6 (4.5–33.3)	5.7 (0.02^+^–14.1)	10.3 (4.1–13.0)	8.3	25.0	58.3	91.7	25.0
		Arm B (*n* = 15)	46.7	86.7	73.3	NR	NR	19.2 (7.6–35.1^+^)	6.8 (0.9^+^–24.6^+^)	5.8 (3.0‐)	0	46.7	46.7	93.3	28.6
		Arm C (*n* = 15)	46.7	60.0	40.0	NR	NR	14.9 (3.2–34.2^+^)	4.8 (0.7–28.7^+^)	5.5 (2.8‐)	0	46.7	26.7	73.3	42.9
		Arm D (*n* = 14)	42.9	85.7	50.0	NR	NR	(8.8–30.1^+^)	7.1 (0.02^+^–24.8^+^)	19.6 (5.1‐)	0	42.9	42.9	85.7	66.7
2017	Hellmann et al. 33	Total (*n* = 77)	42.9	NR	NR	42.9	35.1	NR	NR	NR	0	42.9	24.7	67.5	75.6
		Arm A (*n* = 38)	47.4	NR	NR	81.6	36.8	NR	8.1 (5.6–13.6)	(11.3‐)	0	47.4	31.6	78.9	72.2
		Arm B (*n* = 39)	38.5	NR	NR	71.8	33.3	NR	3.9 (2.6–13.2)	(8.4‐)	0	38.5	17.9	56.4	80.0
2016	Nishio et al. 34	Total (*n* = 76)	25.0	68.0	NR	84.2	22.4	17.1 (13.3–23.0)	2.8 (1.4–3.4)	(1.6–29.1)	1.3	23.7	22.4	NR	NR
2017	Carbone et al. 35	Total (*n* = 541)													
		Nivolumab (*n* = 270)	26.0	56.0	40.0	71.0	18.0	14.4 (11.7–19.4)	4.2 (3.0–5.6)	12.1 (1.7–19.4 + )	2.0	24.0	38.0	NR	NR
		Platinum chemotherapy (*n* = 271)	33.0	54.0	47.0	92.0	51.0	13.2 (10.7–21.0)	5.9 (5.4–6.9)	5.7 (1.4–21.0 + )	0	33.0	47.0	NR	NR

ORR, Objective response rate; 1‐yOS rate, 1‐year overall survival rate; PFS at 24‐week rate, progression‐free survival rate at 24 weeks; any‐grade AEs, any‐grade treatment‐related adverse effects; Grade 3–4 AEs, grade 3–4 treatment‐related adverse effects; mOS, median overall survival; mPFS, median progression‐free survival; mDOR, median duration of response; CR, complete response; PR, partial response; SD, stable disease; DCR, disease control rate; NR, no relevant statistic data.

a Only range (one or two sides) available.

b Only median available.

c Median (range).

### Quality assessment

The three RCTs 23, 24, 35 were assessed using the Cochrane risk of bias tool and did not demonstrate allocation concealment and blinding methods, but generated random sequences, provided complete outcome data, reported no selective outcome, and were free of other bias (Table S2). We included noncomparative studies from top journals or distinguished scientists, which ensured their high quality and integrity.

### Main outcome

In 19 studies reporting ORR 16, 17, 18, 19, 20, 21, 22, 23, 24, 25, 26, 27, 28, 30, 31, 32, 33, 34, 35, the pooled ORR was 20% (95% CI: 16–24%) with high heterogeneity (*I* ^2^ = 87.3%, *P *<* *0.001). After omitting six studies with large heterogeneity 21, 28, 32, 33, 34, 35, the pooled ORR was 18% (95% CI: 15–20%) with low heterogeneity (*I* ^2^ = 20.6%, *P *=* *0.235; Table 3).

**Table 3:** Pooled objective response rate (ORR) and modified objective response rate (ORR) in non‐small‐cell lung cancer (NSCLC) patients for included studies

Study		ORR (predeleted)		Study		ORR (postdeleted)	
		Median	95% CI			Median	95% CI
	Total	20%	16–24%		Total	18%	15–20%
2012	Topalian et al.	18%	10–27%	2012	Topalian et al.	18%	10–27%
2013	Rizvi et al.	32%	18–46%	2013	Rizvi et al.	32%	18–46%
2014	Antonia et al.	16%	6–26%	2014	Antonia et al.	16%	6–26%
2014	Antonia et al.	22%	10–34%	2014	Antonia et al.	22%	10–34%
2014	Ramalingam et al.	12%	5–18%	2014	Ramalingam et al.	12%	5–18%
2014	Rizvi et al.	9%	0–19%	2015	Bauer et a	14%	9–18%
2015	Bauer et a	14%	9–18%	2015	Borghaei et al.	19%	15–24%
2015	Borghaei et al.	19%	15–24%	2015	Brahmer et al.	20%	13–27%
2015	Brahmer et al.	20%	13–27%	2015	Gettinger et al.	17%	11–24%
2015	Gettinger et al.	17%	11–24%	2015	Nishio et al.	22%	14–29%
2015	Nishio et al.	22%	14–29%	2015	Rizvi et al.	14%	8–21%
2015	Rizvi et al.	14%	8–21%	2016	Crino et al.	18%	14–22%
2016	Bidoli et al.	4%	1–7%	2016	Gettinger et al.	23%	12–35%
2016	Crino et al.	18%	14–22%	Overall (*I* ^2^ * *= 20.6%, *P *=* *0.235); Egger's test (*P *=* *0.098)			
2016	Gettinger et al.	23%	12–35%				
2016	Rizvi et al.	43%	30–56%				
2017	Hellmann et al.	43%	32–54%				
2016	Nishio et al.	25%	15–35%				
2017	Carbone et al.	26%	21–31%				
Overall (*I* ^2^ * *= 87.3%, *P *<* *0.001); Egger's test (*P *=* *0.002)							

Ten studies reported the 1‐yOS rate 21, 23, 24, 25, 27, 29, 31, 32, 34, 35; the pooled 1‐yOS rate was 55% (95% CI: 48–63%) with high heterogeneity (*I* ^2^ = 83.6%, *P *<* *0.001). After omitting five studies with great heterogeneity 21, 31, 32, 34, 35, the pooled 1‐yOS rate was 45% (95% CI: 40–50%) with low heterogeneity (*I* ^2^ = 44.2%, *P* = 0.128; Table 4).

**Table 4:** Pooled 1‐year overall survival rate (1‐yOS rate) and modified 1‐year overall survival rate (1‐yOS rate) in non‐small‐cell lung cancer (NSCLC) patients for included studies

Study		1‐yOS rate (predeleted)		Study		1‐yOS rate (postdeleted)	
		Median	95% CI			Median	95% CI
	Total	55%	48–63%		Total	45%	40–50%
2014	Rizvi et al.	75%	51–99%	2015	Borghaei et al.	51%	45–57%
2015	Borghaei et al.	51%	45–57%	2015	Brahmer et al.	42%	34–51%
2015	Brahmer et al.	42%	34–51%	2015	Gettinger et al.	42%	33–50%
2015	Gettinger et al.	42%	33–50%	2015	Rizvi et al.	39%	30–48%
2015	Rizvi et al.	39%	30–48%	2016	Brustugun et al.	50%	37–63%
2016	Brustugun et al.	50%	37–63%	Overall (*I* ^2^ = 44.2%, *P *=* *0.128); Egger's test (*P *=* *0.433)			
2016	Gettinger et al.	73%	61–85%				
2016	Rizvi et al.	71%	61–83%				
2016	Nishio et al.	68%	58–78%				
2017	Carbone et al.	56%	50–62%				
Overall (*I* ^2^ = 83.6%, *P *<* *0.001); Egger's test (*P *=* *0.257)							

Six studies reported the PFS at 24 weeks rate 16, 18, 20, 31, 32, 35; the pooled PFS at 24 weeks rate was 37% (95% CI: 30–45%) with moderate heterogeneity (*I* ^2^ = 74.5%, *P *=* *0.004). After omitting two studies with great heterogeneity 16, 20, the pooled result was 42% (95% CI: 37–48%) with quite low heterogeneity (*I* ^2^ = 12.7%, *P *=* *0.329; Table S3).

Fifteen studies reported the any‐grade AEs% 19, 20, 21, 22, 23, 24, 25, 27, 29, 30, 31, 32, 33, 34, 35; the pooled result was 60% (95% CI: 46–75%) with high heterogeneity (*I* ^2^ = 98.0%, *P *<* *0.001). After omitting two studies with tremendous heterogeneity 22, 32, the pooled results were 61% (95% CI: 50–73%) with little reduction in heterogeneity (*I* ^2^ = 96.4%, *P *<* *0.001; Table 5).

**Table 5:** Pooled any‐grade adverse effects rate (any‐grade AEs%) and modified any‐grade adverse effects rate (any‐grade AEs%) in non‐small‐cell lung cancer (NSCLC) patients for included studies

Study		Any‐grade AEs% (predeleted)		Study		Any‐grade AEs% (postdeleted)	
		Median	95% CI			Median	95% CI
	Total	60%	46–75%		Total	61%	50–73%
2014	Antonia et al.	85%	74–95%	2014	Antonia et al.	85%	74–95%
2014	Ramalingam et al.	71%	63–79%	2014	Ramalingam et al.	71%	63–79%
2014	Rizvi et al.	73%	58–88%	2014	Rizvi et al.	73%	58–88%
2015	Bauer et al.	13%	9–18%	2015	Borghaei et al.	68%	63–74%
2015	Borghaei et al.	68%	63–74%	2015	Brahmer et al.	58%	50–66%
2015	Brahmer et al.	58%	50–66%	2015	Gettinger et al.	41%	33–50%
2015	Gettinger et al.	41%	33–50%	2015	Rizvi et al.	74%	66–82%
2015	Rizvi et al.	74%	66–82%	2016	Brustugun et al.	31%	19–43%
2016	Brustugun et al.	31%	19–43%	2016	Crino et al.	29%	24–33%
2016	Crino et al.	29%	24–33%	2016	Gettinger et al.	71%	59–84%
2016	Gettinger et al.	71%	59–84%	2017	Hellmann et al.	43%	32–54%
2016	Rizvi et al.	95%	89–100%	2016	Nishio et al.	84%	76–92%
2017	Hellmann et al.	43%	32–54%	2017	Carbone et al.	71%	66–75%
2016	Nishio et al.	84%	76–92%	Overall (*I* ^2^ * *= 96.4%, *P *<* *0.001); Egger's test (*P *=* *0.367)			
2017	Carbone et al.	71%	66–75%				
Overall (*I* ^2^ * *= 98.4%, *P *<* *0.001); Egger's test (*P *=* *0.175)							

Eighteen studies reported grade 3–4 AEs% 17, 18, 19, 20, 21, 22, 23, 24, 25, 26, 27, 29, 30, 31, 32, 33, 34, 35; the pooled grade 3–4 AEs% was 20% (95% CI: 15–25%) with high heterogeneity (*I* ^2^ = 93.4%, *P *<* *0.001). After omitting five studies with extraordinary heterogeneity 17, 18, 19, 32, 33, the pooled result was 12% (95% CI: 9–16%) with little reduction in heterogeneity (*I* ^2^ = 89.1%, *P *<* *0.001; Table 6). Table S4 presents more details on the AEs%.

**Table 6:** Pooled grade 3–4 adverse effects rate (grade 3–4 AEs%) and modified grade 3–4 adverse effects rate (grade 3–4 AEs%) in non‐small‐cell lung cancer (NSCLC) patients for included studies

Study		ORR (predeleted)		Study		ORR (postdeleted)	
		Median	95% CI			Median	95% CI
	Total	20%	15–25%		Total	11%	7–14%
2013	Rizvi et al.	49%	34–64%	2014	Ramalingam et al.	15%	9–21%
2014	Antonia et al.	49%	35–63%	2014	Rizvi et al.	25%	10–40%
2014	Antonia et al.	45%	31–59%	2015	Bauer et al.	2%	0–4%
2014	Ramalingam et al.	15%	9–21%	2015	Borghaei et al.	10%	7–14%
2014	Rizvi et al.	25%	10–40%	2015	Brahmer et al.	7%	3–11%
2015	Bauer et al.	2%	0–4%	2015	Gettinger et al.	14%	8–20%
2015	Borghaei et al.	10%	7–14%	2015	Nishio et al.	16%	9–23%
2015	Brahmer et al.	7%	3–11%	2015	Rizvi et al.	17%	10–24%
2015	Gettinger et al.	14%	8–20%	2016	Brustugun et al.	5%	0–11%
2015	Nishio et al.	16%	9–23%	2016	Crino et al.	5%	3–8%
2015	Rizvi et al.	17%	10–24%	2016	Gettinger et al.	19%	8–30%
2016	Brustugun et al.	5%	0–11%	Overall (*I* ^2^ * *= 86.5%, *P* < 0.001); Egger's test (*P *<* *0.001)			
2016	Crino et al.	5%	3–8%				
2016	Gettinger et al.	19%	8–30%				
2016	Rizvi et al.	45%	32–58%				
2017	Hellmann et al.	35%	24–48%				
Overall (*I* ^2^ * *= 93.4%, *P* < 0.001); Egger's test (*P *<* *0.001)							

### Other outcomes

In view of the limitations of clinical meaning and statistical methodology, we elected not to pool the other outcomes.

### Subgroup analysis

To determine whether the treatment effect of nivolumab was consistent across various subgroups, the between‐group treatment effect for the modified ORR and grade 3–4 AEs% was estimated within each category of the following classification variables: study design (RCT, noncomparative open‐label cohort study), medication (nivolumab, nivolumab with other drugs), program subgroup (no subgroup therapy, with subgroup therapy), region (North America, Europe), study phase (I, II, III), and histology (squamous, nonsquamous, mixed). Table 7 lists the subgroup analysis results.

**Table 7:** Subgroup analysis of modified objective response rate (ORR) and grade 3–4 adverse effects rate (grade 3–4 AEs%) of nivolumab in non‐small‐cell lung cancer (NSCLC) patients

Group	ORR				Grade 3–4 AEs%			
	No. of studies	ES (95% CI)	*P* heterogeneity	*I* ^2^ (%)	No. of studies	ES (95% CI)	*P* heterogeneity	*I* ^2^ (%)
Total	13	18% (15–20%)	0.235	20.6	13	12% (9–16%)	<0.001	89.1
Study design								
Randomized open‐label study	2	19% (16–23%)	0.374	0.0	3	12% (6–18%)	0.002	84.0
Noncomparative open‐label cohort study	11	17% (15–20%)	0.192	26.4	10	13% (8–17%)	<0.001	88.7
Medication type								
Nivolumab	10	17% (15–19%)	0.352	9.9	12	12% (8–16%)	<0.001	89.4
Nivolumab with other drugs^a^	3	22% (13–31%)	0.183	41.4	1	25% (10–40%)	NR	NR
Program subgroup								
Subgroup^b^ therapy	5	19% (15–24%)	0.356	8.9	2	17% (7–27%)	0.176	45.3
No subgroup therapy	8	17% (15–19%)	0.195	29.2	11	12% (8–16%)	<0.001	89.8
Region								
North America	12	18% (15–20%)	0.185	26.4	11	14% (9–19%)	<0.001	90.7
Europe	1	18% (14–22%)	NR	NR	2	5% (3–8%)	0.949	0.0
Study phase								
I	7	18% (14–22%)	0.199	30.1	4	14% (3–24%)	<0.001	84.6
II	3	16% (10–21%)	0.145	48.3	4	17% (13–21%)	0.638	0.0
III	3	19% (16–22%)	0.872	0.0	5	9% (5–13%)	<0.001	84.6
Histology								
Squamous	4	16% (13–20%)	0.231	30.2	4	10% (5–16%)	0.001	81.3
Nonsquamous	1	19% (15–24%)	NR	NR	2	16% (4–27%)	0.018	82.2
Mixed histology^c^	8	19% (15–22%)	0.203	28.3	7	13% (6–20%)	<0.001	92.1

ES, Effect size (main outcome such as ORR or Grade 3–4 AEs% with corrected standard deviation); NR, no relevant statistic data.

a Ipilimumab, bevacizumab, platinum‐based doublet chemotherapy (gemcitabine, cisplatin, pemetrexed, carboplatin, paclitaxel).

b The study contained different nivolumab therapy strategy of various dosage and duration et al.

c Squamous, nonsquamous, adenocarcinoma, unknown types etc.

### ORR in PD‐L1 expression

**Figure 2:**
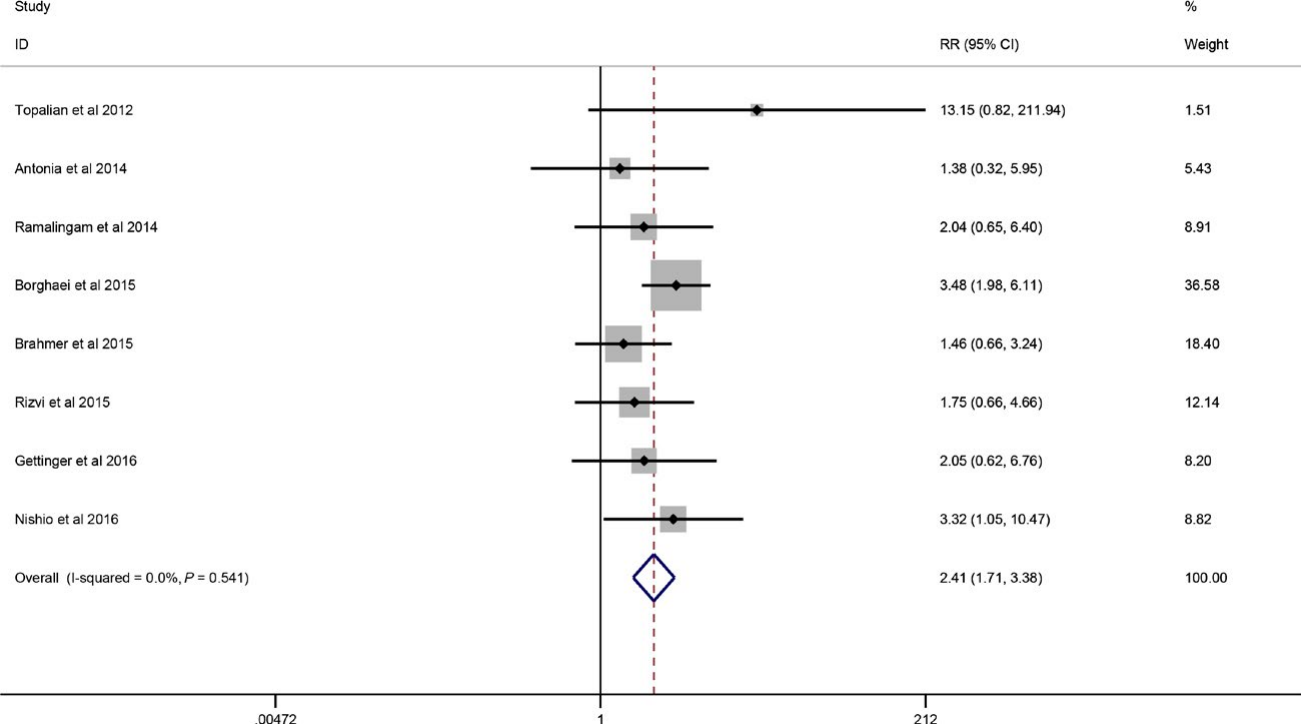
Forrest plots showing comparison of ORR between positive and negative PD‐L1 expression in a random‐effects model.

**Figure 3:**
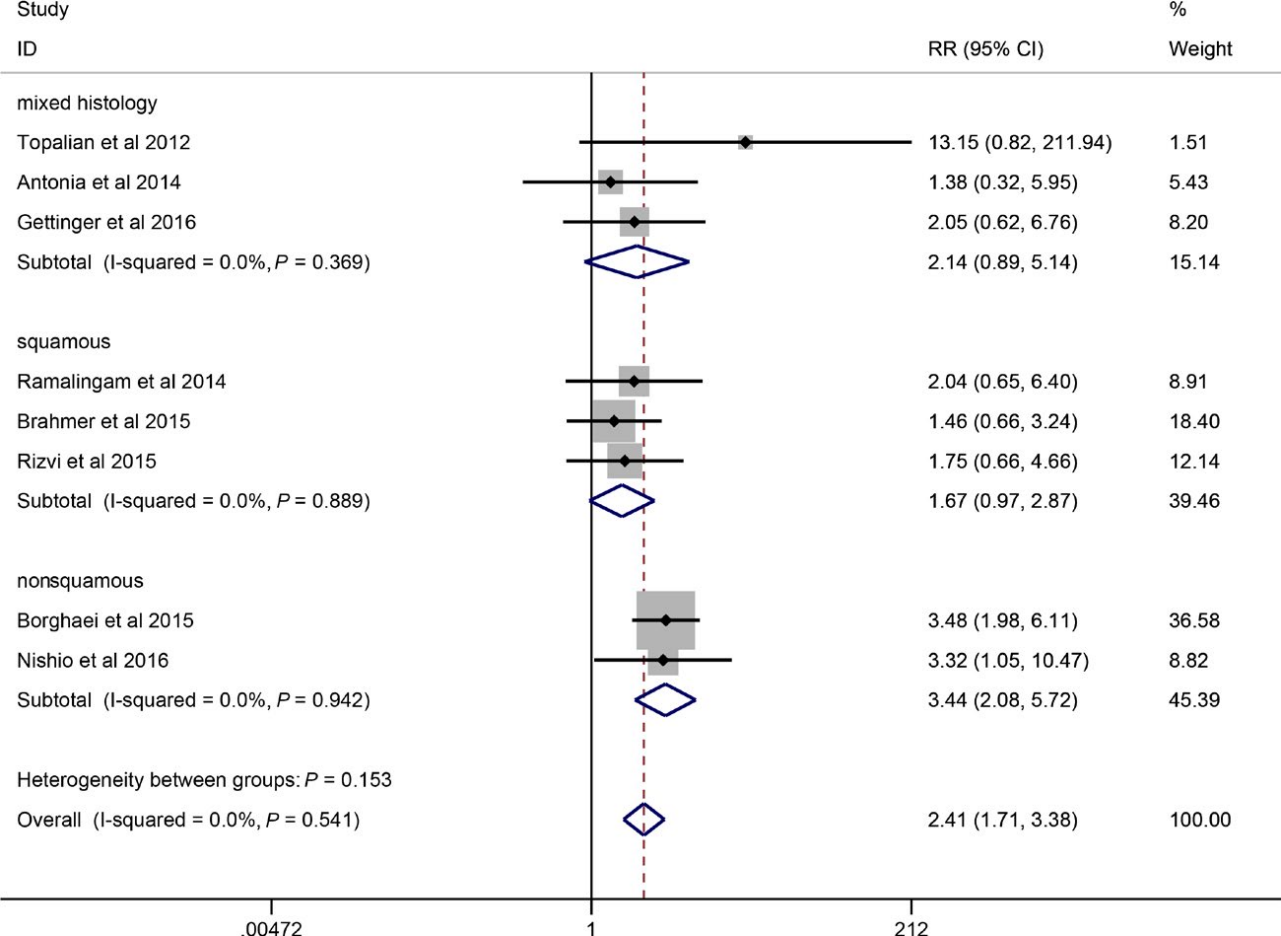
Forrest plots showing subgroup comparison of ORR between positive and negative PD‐L1 expression in a random‐effects model.

Eight studies 16, 18, 20, 23, 24, 27, 31, 34 reported the pending relationship between ORR and PD‐L1 expression systematically. An expression level of <5% was deemed PD‐L1‐negative, and ≥5% expression was deemed PD‐L1‐positive. RR with the random‐effects model was used because its assumptions accounted for the presence of variability among the studies. ORR had significant associations with PD‐L1‐positive and PD‐L1‐negative expression (95% CI: 1.71–3.38, *P *<* *0.001) with no heterogeneity (*I* ^2^ = 0, *P *=* *0.541; Fig. 2). Specifically, there was no significant association between PD‐L1 expression with mixed histology (95% CI: 0.89–5.14, *P *=* *0.089) and squamous NSCLC (95% CI: 0.97–2.87, *P *=* *0.066) with no heterogeneity (*I* ^2^ = 0, *P *=* *0.369; *I* ^2^ = 0, *P *=* *0.889; Fig. 3).

### Publication bias diagnosis

The Egger regression test suggested bias in the ORR and grade 3–4 AEs% analysis and that the bias remained present in the grade 3–4 AEs% after studies of large variability had been removed. In fact, some deviations remained in the Egger test, as large heterogeneity across studies contributes to false positives in diagnosing publication bias 36. Furthermore, most of the included clinical studies had been sponsored by BMS, and there is a distinct possibility that some reports associated with inverse positive results had been denied publication due to the desire for profit and pursuit of the sensational benefits of nivolumab. Next, ethical concerns and the particularity of patients hindered the measurement of some indicators; for example, for the 1‐yOS rate, a precise experimental outcome could not be obtained from patients with short life expectancy, and patients with severe AEs in ongoing clinical studies might have discontinued treatment or died, hindering the extensive examination of grade 3–4 AEs%. Moreover, some of the included studies potentially described relevant data selectively (they might have obscured quite serious AEs), resulting in reports of outcome indicators that were not comprehensive and were incomplete. Partial withdrawal or loss of participants, obscured experiment outcomes, and unpublished studies accounted for publication bias.

### Summary

This was a comprehensive single‐arm meta‐analysis of 17 noncomparative open‐label cohort studies and three randomized open‐label studies. The main outcome analysis of nivolumab in advanced NSCLC indicated a satisfactory, durable response with tolerable, manageable AEs. According to present synthesis data, PD‐L1 expression highlighted the different efficacy of nivolumab in patients with different tumor microenvironment. Nivolumab can be regarded as potential antitumor drug and is worth popularization.

## Discussion

From our manuscript we knew, nivolumab showed astonishing and persistent efficacy as well as manageable toxicity. The ORR was closely associated with PD‐L1 expression in that positive PD‐L1 expression resulted in a higher ORR. Most subgroups yielded results consistent with the overall outcome. There was pronounced ORR improvement in patients with nonsquamous NSCLC who had been treated with nivolumab plus ipilimumab; however, concurrent drug use might increase the potential risk of grade 3–4 AEs%. Generally, the RCTs reported better results than the noncomparative studies, and phase III clinical studies reported more precise indications. However, considerable heterogeneity across studies limited the actual efficacy and possibility of first‐line chemotherapy with nivolumab.

Our study is meaningful and has large impact on clinical practice especially in the patients with NSCLC immunotherapy treatment and management. Nivolumab has more beneficial response rate and overall survival than other second‐line chemotherapy drugs (docetaxel), and less adverse effects (AEs) were detected in nivolumab group than in platinum‐based group. All the efficacy and safety profile showed potential promotion of nivolumab. When comparing to other checkpoints (CTLA‐4) inhibitor antibody, the final conclusion still keeps debatable and unclear; however, we found nivolumab plus ipilimumab improved the efficacy of single‐agent nivolumab without significant AEs difference. Based on this finding, concurrent immunotherapy (such as nivolumab plus ipilimumab) might become standard strategy of crucial efficacy in oncology treatment. Furthermore, no evidence showed large exposure of nivolumab enhanced the overall survival of mixed‐histology patients with NSCLC, and treatment‐related AEs kept no significant as well. So according to cost‐effective analyses, nivolumab of 3 mg/kg Q2W is now the well‐accepted adminstration methods. These meanings represent valuable priority for further studies and researches.

Inducing or potentiating immune responses via immunotherapeutic manipulation is a viable treatment approach in lung cancer. Currently, T‐cell modulating agents are being investigated, where antibodies against CTLA‐4, such as ipilimumab, exhibit great efficacy in treating NSCLC in combination with platinum‐based chemotherapy 37. PD‐1 checkpoint blockade highlights the potential efficacy of the broad applicability of immunotherapy, and nivolumab, a BMS‐sponsored human IgG4 monoclonal antibody, is the first approved immunotherapy drug for advanced squamous NSCLC 38. Many reviews 6, 39, 40, 41, 42 and clinical studies have summarized the good tolerance and favorable clinical activity of nivolumab against a wide variety of malignancies, including NSCLC. Nivolumab can block the activated phosphatidylinositol 3‐kinase (PI3K)/AKT phosphatase SHP2 pathway that switches off T‐cell and B‐cell antitumor activity when PD‐1 binds to PD‐L1 43, 44. Some additional signaling pathway may also be involved in the inhibitor process. The encouraging efficacy can also be explained by the phenomenon that when PD‐1 connects to PD‐L1, coding somatic mutations create new epitopes or peptides identified by peripheral immune system to enhance antitumor immunity 45, 46, 47, 48, 49. In the present meta‐analysis, we found that patients with epidermal growth factor receptor (*EGFR*) mutation or anaplastic lymphoma kinase (*ALK*) translocation received additional tyrosine kinase inhibitor therapy, as *EGFR* and *ALK* mutations derive great benefit from nivolumab 50. The included studies involved various dosages and cycles of nivolumab, but the actual benefit and risk remain unknown; some reports have stated that high‐dose nivolumab may confer obvious benefit but with uncontrolled AEs 51, but, on the contrary, some scientists insisted different nivolumab exposure was not associated with patients OS and toxicity 52. The present meta‐analysis shows that the most common AEs caused by nivolumab were fatigue and rash; there were few grade 3–4 AEs in the total AEs. We proved that PD‐L1 expression predicted the efficacy of nivolumab treatment; in addition, high PD‐L1 expression might inhibit tumor differentiation 53.

Although our main outcome analysis on nivolumab treatment is biologically plausible, the results of the included individual studies were dissimilar, as reflected in the significant heterogeneity. Although we selectively removed studies of great variability, heterogeneity remained for any‐grade AEs% and grade 3–4 AEs%. We also performed subgroup analysis, and there was no significant heterogeneity change in grade 3–4 AEs%. Besides study design, the clinical study phase, region, and some unidentified elements also confounded our final outcome.

Differing participant characteristics may cause inconsistent results, and nonuniform, patient‐level *EGFR* mutation or *ALK* translocation could also have led to potential heterogeneity. Not all patients that required ALK inhibitor and EGFR tyrosine kinase inhibitor therapy received it, and most of the studies did not report the details of the administration of the target drugs.

Differing medication administration strategies may also have contributed to the heterogeneity. For example, high‐dose or long cycles of nivolumab may confer more benefit on patients with advanced NSCLC; the concurrent use of nivolumab with platinum‐based drugs potentially increases grade 3–4 AEs%. There is no evidence that intravenous injection can help amplify nivolumab efficacy and reduce the AEs.

Methodologically, to a great extent, a single‐arm meta‐analysis is subject to subjectivity and heterogeneity. Furthermore, the measurement of outcome indicators differed greatly in the studies supported by BMS. Some negative results that would have affected the total efficacy of nivolumab might not have been reported. The lack of a standardized approach for assessing PD‐L1 remained a limitation of the included studies.

Although there was inevitable heterogeneity in the included studies, our meta‐analysis still has some strengths: We included more large‐sample, high‐quality studies, and our results are more convincing than those of Huang et al. 54, who reported a small meta‐analysis that omitted studies on different nivolumab dosages (except 3 mg/kg) and concurrent drug use. We found nivolumab not only demonstrated encouraging ORR but also exhibited durable response rate, longer PFS. More powerful evidence enhanced the beneficial profile of nivolumab and expanded future potential efficacy. However, limited by a few heterogeneity, we still warranted more highly described RCTs. In addition, the present meta‐analysis systematically reviewed the latest published studies, included subgroups of different aspects, and reported the general any‐grade AEs%. Nivolumab has demonstrated extensive activity both as a single agent and in combination with other drugs, and further exploration is worthwhile. PD‐L1 can be a potential biomarker for predicting prognosis in NSCLC, but the benefit of nivolumab is greatly limited in patients with negative PD‐L1 expression 55. Does nivolumab still play a role in patients with advanced NSCLC with brain or bone metastasis?Whether we can find more surrogate checkpoint molecules expressed on resting or activation T cells, natural killers (NK) cells, etc., then reinforce these checkpoints as supplementary indicators for patients’ prognosis and emerging tactics to treat refractory malignants 56? In addition, it will be important to establish the optimal timing of therapy and to determine whether immunotherapies are most effective when used alone or in combination with other agents. Promising results for immunotherapy have been reported, demonstrating CR and cures in patients with aggressive malignancies. The complex AEs and cost of engineering and administering of some forms of immunotherapy limit the use to a distinct patient population. Accordingly, high‐throughput and cost‐effective techniques are being used to broaden the applications of immunotherapy for treating cancer 42, 57.

Our study still has some limitations. Initially, only two small studies reported results for nivolumab as compared with docetaxel; most of the included studies lacked control therapies. Due to the lack of terms of concrete control strategy, we only evaluated the efficacy and risk under subjectivity and selection bias without significantly statistical conclusions. Second, nonuniform patient level and trial level contributed to significant heterogeneity, and partial publication bias undermined the credibility of our results to an extent. However, most confounding factors were derived from methodology restrictions. Lastly, we could not extract sufficient details on the relationship between ORR and PD‐L1 expression; more data are required to support the results of the 1‐yOS rate and PFS at 24 weeks rate and to establish the assessments of other clinical endpoints such as the median OS, median PFS, CR, PR, and SD.

In summary, nivolumab has the potential to mount an ongoing, dynamic immune response for an extended period rather than a temporary killing of tumor cells after the pretarget therapy has been administered. The novel benefits and low risk of AEs enable nivolumab to emerge as a second‐line chemotherapy drug, and with the use of other concurrent checkpoint antibodies, will form a new approach to treating cancer. More high‐quality and adequately powered RCTs are warranted to help interpret these conclusions with caution.

## Conflict of Interest

The authors declare no potential conflict of interests.

## Supporting information


**Table S1.** PRISMA checklist.

**Table S2.** Risk of bias assessments for two randomized studies.

**Table S3.** PFS at 24 weeks rate.

**Table S4.** Detailed treatment‐related adverse effects of nivolumab in non‐small cell lung cancer (NSCLC) patients.
