# Supplementary material for: The benefit and risk of nivolumab in non‐small‐cell lung cancer: a single‐arm meta‐analysis of noncomparative clinical studies and randomized controlled trials
Source: Cancer Med. 2018 Mar 23;7(5):1642–59. doi: 10.1002/cam4.1387 (PMC5943422; doi:10.1002/cam4.1387)
Supplement: Supplementary file 1 — Table S1. PRISMA checklist. [file CAM4-7-1642-s001.docx]

| **Section/topic** | **#** | **Checklist item** | **Reported on page #** |
| --- | --- | --- | --- |
| **TITLE** | | | 1 |
| Title | 1 | The Benefit and Risk of Nivolumab in Non–small Cell Lung Cancer: A Single-arm Meta-analysis of Non-comparative Clinical Studies and Randomized Controlled Trials | 1 |
| **ABSTRACT** | | | 2 |
| Structured summary | 2 | Nivolumab is a programmed cell death 1 (PD-1) receptor inhibitor antibody that enhances immune system anti-tumor activity. Although it is used for treating advanced non–small cell lung cancer (NSCLC), its actual efficacy has not been determined. We searched PubMed, the Cochrane Library, Embase, MEDLINE, and Web of Science for related non-comparative clinical studies and randomized controlled trials (RCTs) to assess nivolumab benefit and risk in NSCLC. The main outcomes were objective response rate (ORR), 1-year overall survival rate (1-yOS rate), and progression-free survival rate at 24 weeks (PFS at 24wks rate), any-grade adverse effects rate (any-grade AEs%), and grade 3–4 AE rate (grade 3–4 AEs%). Relative risk (RR) was used to compare ORR in patients with positive and negative PD-1 ligand 1 (PD-L1) expression. Random-effects models were used to determine pooled effect size and two-sided 95% confidence intervals (95% CI). We included 18 studies (16 non-comparative open-label cohort studies, two RCTs) involving 2787 patients in our meta-analysis. The modified nivolumab ORR was 18% (95% CI: 15%–20%), the 1-yOS rate was 45% (95% CI: 40%–50%), PFS at 24wks rate was 45% (95% CI: 37%–54%), any-grade AEs% was 58% (95% CI: 46%–71%), and grade 3–4 AEs% was 11% (95% CI: %–14% PD-L1 expression was related with the nivolumab ORR. Nivolumab potentially causes ongoing response, long-term PFS, and reduced treatment-related AEs. PD-L1 expression predicts the outcome of nivolumab immunotherapy. More high-quality and well-designed RCTs with large sample sizes are warranted to prove our findings. | 2 |
| **INTRODUCTION** | | | 3-4 |
| Rationale | 3 | Many single arm studies reported the benefit and risk of nivolumab in NSCLC but the results they conveyed was controversial. | 3 |
| Objectives | 4 | Thus we systematically reviewed current available literature to conduct present single arm meta-analysis aiming to describe the benefit and risk of nivolumab in general. The secondary objective was to evaluate whether the objective response rate (ORR) of treated patients with positive and negative PD-L1 expression is significantly difference. | 3-4 |
| **METHODS** | | | 4-5 |
| Protocol and registration | 5 | Our meta-analysis was accordance with PRISMA (Preferred Reporting Items for Systematic Review and Meta-Analysis) and this meta-analysis has been registered in PROSPERO (International Prospective Register of Systematic Reviews, CRD42017064411). | 4 |
| Eligibility criteria | 6 | 1. Adult patients with advanced NSCLC whose life expectancy was at least 3 months and without any autoimmune disease; Eastern Cooperative Oncology Group performance status was ≤2; there were no restrictions and no significant difference on sex, race, region, nationality, pre-treatment; 2. Single-agent nivolumab or in combination with other chemotherapy drugs; 3. Whether comparison had been performed; 4. The main study outcome directly or indirectly included ORR, 1-year overall survival rate (1-yOS rate), progression-free survival rate at 24 weeks (PFS at 24wks rate), any-grade adverse effects rate (any-grade AEs%), and grade 3–4 adverse effects rate (grade 3–4 AEs%) [treatment-related AE status was assessed using the Lung Cancer Symptom Scale and the European Quality of Life–5 Dimensions questionnaire ([9](#_ENREF_9))]; 5. Non-comparative clinical studies (non-comparative open-label studies) and RCTs. | 4-5 |
| Information sources | 7 | We searched PubMed, Cochrane Library, EMBASE, Medline, and the Web of Science for relevant studies published between Jan 1, 2012 to Dec 31, 2017 No language restrictions were applied. | 4-5 |
| Search | 8 | The complete search we took for pubmed went:(Carcinoma, Non-Small-Cell Lung[MeSH Terms] OR Carcinoma, Non Small Cell Lung[Text Word] OR Carcinomas, Non-Small-Cell Lung[Text Word] OR Lung Carcinoma, Non-Small-Cell[Text Word] OR Lung Carcinomas, Non-Small-Cell[Text Word] OR Non-Small-Cell Lung Carcinomas[Text Word) AND (nivolumab[MeSH Terms] OR MDX-1106[Text Word] OR ONO-4538[Text Word] OR BMS-936558[Text Word] OR Opdivo[Text Word]). We also hand-searched reference lists of retrieved literature for further eligible articles. | 4-5 |
| Study selection | 9 | The most complete and novel reports could be included for data extraction and assessments if the objects were duplicated. We excluded review without original data, meta-analysis, animal experiment. | 5 |
| Data collection process | 10 | Two independent investigators (W.X. Zhang and D.L. Yu) reviewed research titles and abstracts, and studies that appeared to be eligible were retrieved for a full-text assessment. Disagreements were resolved by a third investigator (Y.P. Wei). | 5-6 |
| Data items | 11 | First author, the publication year, region, number of participants enrolled, participants characteristics, phase of clinical study, the completeness of cohort, the tumor histology, clinical setting, final endpoint, corresponding provided outcome and study design. | 6 |
| Risk of bias in individual studies | 12 | We adopted the “Cochrane Risk of Bias Tool” to evaluate the quality of eligible RCTs; we only assess the quality of non-comparative clinical studies through powered data volume and integrity, distinguished journals and influential writers or teams. | 6 |
| Summary measures | 13 | Pooled ES could assist to evaluate the benefit and risk of nivolumab in general; relative risk (RR) and random-effects model was adopted to estimate whether there was significant difference about ORR in PD-L1 positive and negative expression patients. | 6 |
| Synthesis of results | 14 | To assess statistical heterogeneity between the studies, the Cochran’s chi-square (Q-test) was performed, with a predefined significance threshold of 0.1. | 7 |

Page 1 of 2

| **Section/topic** | **#** | Checklist item | **Reported on page #** |
| --- | --- | --- | --- |
| Risk of bias across studies | 15 | Potential publication bias among main outcome were assessed by Egger linear regression test. | 7 |
| Additional analyses | 16 | The Cochran’s chi-square (Q-test) was performed and the treatment effects were examined according to quality components. | 7-8 |
| **RESULTS** | | | 8-13 |
| Study selection | 17 | See relevant Fig.1 in our study. | 8 |
| Study characteristics | 18 | See relevant Table 1 and Table 2 in our study. | 8 |
| Risk of bias within studies | 19 | See relevant Table S2 in our study. | 8-9 |
| Results of individual studies | 20 | See relevant Table 3-6 and Table S3, S4 in our study. | 9-12 |
| Synthesis of results | 21 | The main analysis of nivolumab in advanced NSCLC provided a satisfying, durable response with tolerable, manageable adverse effects. ORR had close associations with PD-L1 expression that positive expression brought about higher ORR versus negative. | 9-12 |
| Risk of bias across studies | 22 | There was bias in ORR and-grade 3-4 AEs% analysis and the bias still existed in grade 3-4 AEs% when studies of large variability deleted. | 12-13 |
| Additional analysis | 23 | Give results of additional analyses, if done (e.g., sensitivity or subgroup analyses, meta-regression [see Item 16]). | Not available |
| **DISCUSSION** | | | 13*17 |
| Summary of evidence | 24 | The main analysis of nivolumab in advanced NSCLC provided a satisfying, durable response with tolerable, manageable adverse effects. ORR had close associations with PD-L1 expression that positive expression brought about higher ORR versus negative. | 13-14 |
| Limitations | 25 | Initially, only two small studies reported results for nivolumab as compared with docetaxel; most of the included studies lacked control therapies. Based on this, wenly evaluated the efficacy and risk of nivolumab under subjectivity and selection bias without statistical conclusions in terms of a concrete control strategy. Second, non-uniform patient level and trial level contributed to significant heterogeneity, and partial publication bias undermined the credibility of our results to an extent. However, most confounding factors were derived from methodology restrictions. Lastly, we could not extract sufficient details on the relationship between ORR and PD-L1 expression; more data are required to support the results of the 1-yOS rate and PFS at 24wks rate and to establish the assessments of other clinical endpoints such as the median OS, median PFS, CR, PR, and SD. | 16-17 |
| Conclusions | 26 | Nivolumab immunotherapy has the potential to mount an ongoing, dynamic immune response for an extended time rather than a temporary killing of tumor cells after the pre-target therapy has been administered. Novel benefits and low risk of adverse effects enable nivolumab to be emerging second-line chemotherapy drugs. | 17 |
| **FUNDING** | | | 1 |
| Funding | 27 | This research work was supported by the National Natural Science Foundation of China (81560345). | 1 |

*From:*  Moher D, Liberati A, Tetzlaff J, Altman DG, The PRISMA Group (2009). Preferred Reporting Items for Systematic Reviews and Meta-Analyses: The PRISMA Statement. PLoS Med 6(6): e1000097. doi:10.1371/journal.pmed1000097

For more information, visit: **www.prisma-statement.org**.

Page 2 of 2
