# Supplementary material for: The benefit and risk of nivolumab in non‐small‐cell lung cancer: a single‐arm meta‐analysis of noncomparative clinical studies and randomized controlled trials
Source: Cancer Med. 2018 Mar 23;7(5):1642–59. doi: 10.1002/cam4.1387 (PMC5943422; doi:10.1002/cam4.1387)
Supplement: Supplementary file 2 — Table S2. Risk of bias assessments for two randomized studies. [file CAM4-7-1642-s002.docx]

**Table S2.** Risk of bias assessments for two randomized studies

| Study | Adequate sequence generation? | Allocation concealment? | Blinding of participants, personnel, and outcome assessors? | Incomplete outcome data? | No seleative outcome reporting? | Other bias? | Overall risk of bias |
| --- | --- | --- | --- | --- | --- | --- | --- |
|  |  |  |  |  |  |  |  |
| Borghaei et al ([23](#_ENREF_23)) | Adequate | Unadequate | Unadequate | Adequate | Adequate | Adequate | Low |
| Brahmer et al ([24](#_ENREF_24))  Carbone et al (35) | Adequate  Adequate | Unadequate  Unadequate | Unadequate  Unadequate | Adequate  Adequate | Adequate  Adequate | Adequate  Adequate | Low  Low |
