# Supplementary material for: The benefit and risk of nivolumab in non‐small‐cell lung cancer: a single‐arm meta‐analysis of noncomparative clinical studies and randomized controlled trials
Source: Cancer Med. 2018 Mar 23;7(5):1642–59. doi: 10.1002/cam4.1387 (PMC5943422; doi:10.1002/cam4.1387)
Supplement: Supplementary file 3 — Table S3. PFS at 24 weeks rate. [file CAM4-7-1642-s003.docx]

**Table S3.** Pooled progression-free survival rate at 24 weeks (PFS at 24 wks rate) and modified progression-free survival rate at 24 weeks (PFS at 24 wks rate) in non-small cell lung cancer (NSCLC) patients for included studies

| **Study** | | **PFS at 24wks rate (pre-deleted)** | |  | **Study** | | **PFS at 24wks rate (post-deleted)** | |
| --- | --- | --- | --- | --- | --- | --- | --- | --- |
|  |  | **median** | **95% CI** |  |  |  | **median** | **95% CI** |
|  | Total | 37% | 30%-45% |  |  | Total | 42% | 37%-48% |
| 2012 | Topalian et al | 26% | 16%-36% |  | 2014 | Antonia et al | 41% | 28%-55% |
| 2014 | Antonia et al | 41% | 28%-55% |  | 2016 | Gettinger et al | 40% | 27%-54% |
| 2014 | Ramalingam et al | 27% | 19%-35% |  | 2016 | Rizvi et al | 53% | 40%-66% |
| 2016 | Gettinger et al | 40% | 27%-54% |  | 2017 | Carbone et al | 40% | 37%-48% |
| 2016 | Rizvi et al | 53% | 40%-66% |  | Overall (*I^2^* = 12.7%, *p* = 0.329); Egger's test (*P* = 0.460) | | | |
| 2017 | Carbone et al | 40% | 37%-48% |  |  |  |  |  |
| Overall (*I^2^* = 74.5%, *p* = 0.004); Egger's test (*P* = 0.070) | | | |  |  |  |  |  |
|  |  |  |  |  |  |  |  |  |
