# Supplementary material for: The benefit and risk of nivolumab in non‐small‐cell lung cancer: a single‐arm meta‐analysis of noncomparative clinical studies and randomized controlled trials
Source: Cancer Med. 2018 Mar 23;7(5):1642–59. doi: 10.1002/cam4.1387 (PMC5943422; doi:10.1002/cam4.1387)
Supplement: Supplementary file 4 — Table S4. Detailed treatment‐related adverse effects of nivolumab in non‐small cell lung cancer (NSCLC) patients. [file CAM4-7-1642-s004.docx]

**Table S4.** Detailed treatment-related adverse effects of nivolumab in non-small cell lung cancer (NSCLC) patients

| Types of common treatment related AEs^a^ | Total patients | Any-grade AEs% | 95% CI | Grade 3-4 AEs% | 95% CI |
| --- | --- | --- | --- | --- | --- |
|  |  |  |  |  |  |
|  |  |  |  |  |  |
| Fatigue [[7-9](#_ENREF_7), [12](#_ENREF_12), [28](#_ENREF_28), 34, 35] | 992 | 18% | 14%-22% | 6% | 0-13% |
| Rash [[9](#_ENREF_9), [12](#_ENREF_12), [15](#_ENREF_15), [27](#_ENREF_27), [28](#_ENREF_28), 34, 35] | 703 | 13% | 9%-18% | 1% | 0-2% |
| Decreasd appetite [[7](#_ENREF_7), [8](#_ENREF_8), [28](#_ENREF_28), 34, 35] | 882 | 13% | 11%-15% | 0.2% | 0-1% |
| Nausea [[7](#_ENREF_7), [8](#_ENREF_8), [12](#_ENREF_12), [28](#_ENREF_28), 34, 35] | 934 | 12% | 10%-14% | 0.4% | 0-0.8% |
| Diarrhea [[7-9](#_ENREF_7), [12](#_ENREF_12), [15](#_ENREF_15), [27](#_ENREF_27), [28](#_ENREF_28), 34, 35] | 1125 | 11% | 8%-14% | 1.1% | 0.5%-1.8% |
| Asthenia [[7](#_ENREF_7), [8](#_ENREF_8), [28](#_ENREF_28), 35] | 806 | 7% | 0%-13% | 0.2% | 0-0.6% |
| Pneumonitis [[9](#_ENREF_9), [15](#_ENREF_15), [27](#_ENREF_27), [28](#_ENREF_28)] | 308 | 8% | 5%-10% | 4% | 2%-6% |
| Myalgia [[7](#_ENREF_7), [8](#_ENREF_8), [28](#_ENREF_28)] | 539 | 3% | 1%-5% | 0.4% | 0-1% |
| Anemia [[7](#_ENREF_7), [8](#_ENREF_8), [28](#_ENREF_28), 35] | 806 | 3% | 2%-4% | 0.2% | 0-0.5% |
| **Abbreviation**: AE: adverse effects; CI: confident interval ^a^ AEs status are assessed with the use of the "Lung Cancer Symptom Scale and the European Quality of Life–5 Dimensions questionnaire" | | | | | |
|  |  |  |  |  |  |
|  |  |  |  |  |  |
